# Targeted therapies for congenital myasthenic syndromes: systematic review and steps towards a treatabolome

**DOI:** 10.1042/ETLS20180100

**Published:** 2019-01-28

**Authors:** Rachel Thompson, Gisèle Bonne, Paolo Missier, Hanns Lochmüller

**Affiliations:** 1 Institute of Genetic Medicine, Newcastle University, International Centre for Life, Newcastle upon Tyne NE1 3BZ, U.K.; 2 Sorbonne Université, INSERM UMRS 974, Center of Research in Myology, Institut de Myologie, F-75013 Paris, France; 3 School of Computing, Newcastle University, Urban Sciences Building, Firebrick Avenue, Newcastle upon Tyne NE4 5TG, U.K.; 4 Children's Hospital of Eastern Ontario Research Institute, University of Ottawa, Ottawa, Canada; 5 Division of Neurology, Department of Medicine, The Ottawa Hospital, Ottawa, Canada; 6 Department of Neuropediatrics and Muscle Disorders, Medical Center – University of Freiburg, Faculty of Medicine, Freiburg, Germany; 7 Centro Nacional de Análisis Genómico (CNAG-CRG), Center for Genomic Regulation, Barcelona Institute of Science and Technology (BIST), Barcelona, Spain

**Keywords:** bioinformatics, congenital myasthenic syndromes, drug therapy, neuromuscular diseases, systematic review

## Abstract

Despite recent scientific advances, most rare genetic diseases — including most neuromuscular diseases — do not currently have curative gene-based therapies available. However, in some cases, such as vitamin, cofactor or enzyme deficiencies, channelopathies and disorders of the neuromuscular junction, a confirmed genetic diagnosis provides guidance on treatment, with drugs available that may significantly alter the disease course, improve functional ability and extend life expectancy. Nevertheless, many treatable patients remain undiagnosed or do not receive treatment even after genetic diagnosis. The growth of computer-aided genetic analysis systems that enable clinicians to diagnose their undiagnosed patients has not yet been matched by genetics-based decision-support systems for treatment guidance. Generating a ‘treatabolome’ of treatable variants and the evidence for the treatment has the potential to increase treatment rates for treatable conditions. Here, we use the congenital myasthenic syndromes (CMS), a group of clinically and genetically heterogeneous but frequently treatable neuromuscular conditions, to illustrate the steps in the creation of a treatabolome for rare inherited diseases. We perform a systematic review of the evidence for pharmacological treatment of each CMS type, gathering evidence from 207 studies of over 1000 patients and stratifying by genetic defect, as treatment varies depending on the underlying cause. We assess the strength and quality of the evidence and create a dataset that provides the foundation for a computer-aided system to enable clinicians to gain easier access to information about treatable variants and the evidence they need to consider.

## Introduction

Rare genetic disorders are individually uncommon but collectively frequent, affecting as many as 1 in 17 people [1]. While most rare diseases do not currently have curative gene-based therapies, in a small but increasing number of cases, a confirmed genetic diagnosis immediately provides guidance on treatment, in some cases significantly altering the disease course, improving functional ability and quality of life, and extending life expectancy. Marketed therapies or, in some cases, even a commonly available drug may provide effective or even curative therapy by replacing a deficient enzyme or restoring a biological function despite not correcting the genetic code. Examples include metabolic diseases (Gaucher, Fabry, and Pompe) where enzyme replacement therapies are available [2], channelopathies of brain, nerve, neuromuscular junction and muscle where there are effective drugs to prevent brain damage or muscle weakness [3], or inborn errors of metabolism where severe organ damage and intellectual disability can be prevented or reduced through dietary mechanisms or specific vitamin and cofactor replacement [4]. Furthermore, genetic therapies in which the gene itself is not repaired but defective transcription is targeted via read-through therapies or exon skipping have recently been developed and gained marketing authorization for rare neuromuscular diseases such as Duchenne muscular dystrophy [5], amyloid neuropathy [6,7] and spinal muscular atrophy [8]. In other cases, non-pharmacological treatment and follow-up, such as regular cardiac monitoring, or specific interventions, such as implantation of a pacemaker or implantable cardioverter defibrillator, may be indicated owing to the risk a certain mutation confers [9]; or a particular drug may be contraindicated owing to mutation-specific drug interactions or severe adverse events of certain drugs associated with genomic variants such as malignant hyperthermia [10].

Unfortunately, the lack of information and expertise that disproportionately affects rare disease means that not all patients harboring such treatable variants are put onto the appropriate treatment even when their diagnosis is confirmed, meaning that they miss out on receiving the best available care and may suffer harm and potentially avoidable risk to life if the potential for treatment is not recognized and acted upon in a timely way.

As increasing numbers of patients are diagnosed through next-generation sequencing in research projects or within their national healthcare systems, it becomes ever more crucial to enable flagging of potentially treatable cases at a gene or variant level in much the same way as progress has already been made in flagging pathogenicity inferences, so that when a diagnosis is made, the treatment options are immediately accessible to the clinical end-user and the patient rapidly receives the best care. At present, however, this knowledge is largely available only in ‘human-readable’ scientific publications or in expert practice and not captured in computer-accessible form that would allow automatic recognition and flagging in analysis and decision-support systems.

The transformation of human-readable expert knowledge into electronic decision-support systems must begin with a systematic review of the evidence available in order to accurately assess levels of confidence about particular interventions and minimize potential error and bias. If collected with due attention not only to the treatment but also to the genetic background of the participants, the evidence gathered through such a review can be incorporated into a database that serves as an information source for genomic analysis systems and that can provide the input for flagging of potentially treatable variants as described above. This approach, which we have termed the ‘treatabolome’, is the focus of research within projects such as Solve-RD [11] as part of a broader strategy not only to increase diagnostic rates through novel sequencing methodologies but also to increase the impact of diagnosis by increasing the speed with which patients receive the most appropriate treatment for their condition.

In this present work, we aimed to create a proof of concept of the treatabolome by performing a systematic review of a defined set of rare inherited neuromuscular conditions known as the congenital myasthenic syndromes (CMS), many of which are amenable to treatment, and capturing the output of the review in a format suitable for transformation into a computer-readable database.

## The systematic review

### Background

#### Features of CMS

**Figure 1.**
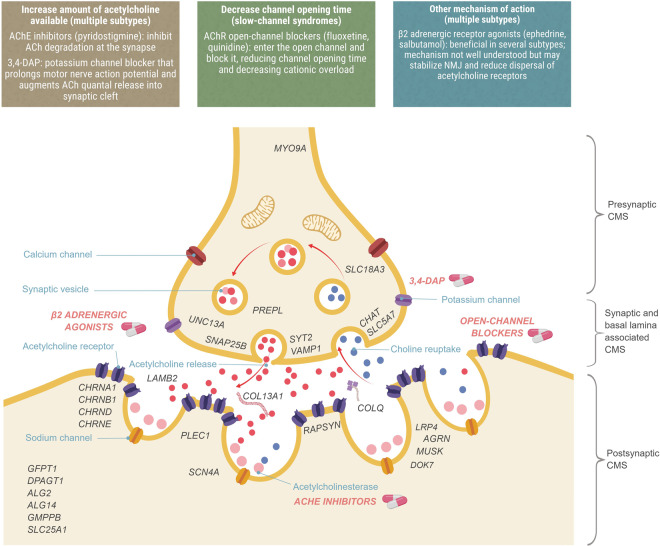
Localization of CMS types and therapeutic strategies. CMS types are stratified according to the location of the genetic defect into presynaptic, synaptic, and basal lamina-associated, postsynaptic and other, and then further stratified by genetic and functional defect. Therapeutic strategies act on different parts of the NMJ and are effective in different types.

CMS are a heterogeneous group of rare inherited neuromuscular disorders characterized by fatigable weakness of skeletal muscle owing to compromised function of the neuromuscular junction (NMJ). The phenotype is caused by failure of transmission across this synapse connecting the nerve with the muscle, whereby an incoming nerve stimulus does not consistently lead to muscle excitation and contraction. Neuromuscular transmission is mediated by the generation of an action potential causing the release of acetylcholine from the nerve terminal into the synaptic cleft, its binding to the acetylcholine receptor (AChR) with the opening of its ion channel and the enzymatic breakdown of acetylcholine by acetylcholinesterase (AChE; Figure 1). Pathophysiological mechanisms acting on any part of this chain and resulting in a reduction in the amount of acetylcholine released, the impairment of the AChR, reduction in the number of receptors or defective breakdown of acetylcholine may lead to CMS. The majority of CMS types are caused by defects in the AChR itself, but they can also result from causative variants affecting presynaptic proteins or proteins associated with the synaptic basal lamina or variants causing defects in endplate development and maintenance or defects in protein glycosylation. Defective neuromuscular transmission presents clinically as fatigable weakness due to increasing impairment of transmission across the NMJ with repeated activation.

The majority of cases of CMS show disease onset within the first year of life, but in some patients, symptoms do not appear until later in childhood or even adulthood. Additional symptoms may include weakness of ocular, facial and bulbar muscles causing ptosis, ophthalmoplegia and feeding difficulties; respiratory difficulties including episodic apnea; and joint contractures. With the advent of next-generation sequencing (NGS), the number of genetic defects reported as causative of a CMS phenotype has increased dramatically, with over 30 genes now implicated [12]. Although all CMS subtypes share the common features of NMJ pathology and fatigable weakness, the severity of the disease, its course of progression, specific phenotypic manifestations and effective treatments are highly variable between subtypes and are often gene- or even mutation-specific.

#### Treatment of CMS

Unlike the related autoimmune disease myasthenia gravis, CMS is not caused by an immune response, and immunomodulating therapies are therefore not effective [13]. Most CMS subtypes are nevertheless amenable to some form of pharmacotherapy, but pharmacological treatment varies by subtype, with the drugs appropriate for one type potentially making another worse [14]. Treatment strategies are illustrated in Figure 1 and Table 1, and are broadly dependent on whether it is beneficial to increase the amount of acetylcholine available in the synaptic cleft (for which commonly administered therapies include AChE inhibitors such as pyridostigmine, which inhibits acetylcholine breakdown, and the potassium-channel blocker 3,4-diaminopyridine, which increases the quantal release of acetylcholine) or to shorten the excessive duration of synaptic current in slow-channel syndromes by reducing the channel-open time (for which the open-channel blockers fluoxetine and quinidine may be used). β_2_ adrenergic receptor agonists such as ephedrine and salbutamol (albuterol) are also widely prescribed for CMS, having serendipitously been discovered in the 1930s to be beneficial in autoimmune myasthenia gravis [13], but despite being first-line treatment in some CMS types, their mechanism of action is not well understood, though it is hypothesized to act as a backup to the agrin complex, stabilizing endplate structures and reversing AChR dispersal [15].

**Table 1:** CMS treatment recommendations stratified by type, including key references and total number of primary reports evaluated

Gene involved	Descriptive name	First-line treatment recommendation	Supplemental treatment recommendation 1	Supplemental treatment recommendation 2	Likely ineffective	Avoid treatment (may worsen)	Expert summary of the evidence	Key reference	Number of publications
*AGRN*	Congenital myasthenic syndrome due to agrin deficiency caused by pathogenic variants in *AGRN*	Salbutamol or ephedrine				Pyridostigmine	Small number of reported cases; exploratory treatment with β2-adrenergic receptor agonists	[56]	7
*ALG14*	Congenital myasthenic syndrome due to a defect of glycosylation caused by pathogenic variants in *ALG14*	Pyridostigmine	3,4-DAP				Small number of reported cases; exploratory treatment with an acetylcholinesterase inhibitor	[57]	2
*ALG2*	Congenital myasthenic syndrome due to a defect of glycosylation caused by pathogenic variants in *ALG2*	Pyridostigmine	3,4-DAP				Small number of reported cases; exploratory treatment with an acetylcholinesterase inhibitor	[57]	1
*CHAT*	Congenital myasthenic syndrome due to endplate choline acetyltransferase deficiency caused by pathogenic variants in *CHAT*	Pyridostigmine	3,4-DAP	Salbutamol or ephedrine			Acetylcholinesterase inhibitors recommended including in oligosymptomatic patients to reduce EA	[58]	18
*CHRNA1*	Slow-channel congenital myasthenic syndrome due to an acetylcholine receptor defect caused by a pathogenic variant in *CHRNA1*	Fluoxetine or quinidine				Pyridostigmine	Channel blocker recommended; avoid acetylcholinesterase inhibitors	[35,42]	8
*CHRNA1*	Fast-channel congenital myasthenic syndrome due to an acetylcholine receptor defect caused by pathogenic variants in *CHRNA1*	Pyridostigmine	Salbutamol or ephedrine	3,4-DAP		Fluoxetine or quinidine	Acetylcholinesterase inhibitors recommended; may require add-on second-line therapy	[59]	4
*CHRNA1*	Congenital myasthenic syndrome due to primary acetylcholine receptor deficiency caused by pathogenic variants in *CHRNA1*	Pyridostigmine	3,4-DAP	Salbutamol or ephedrine			Small number of reported cases; exploratory treatment with acetylcholinesterase inhibitor	[40]	3
*CHRNB1*	Slow-channel congenital myasthenic syndrome due to an acetylcholine receptor defect caused by a pathogenic variant in *CHRNB1*	Fluoxetine or quinidine				Pyridostigmine	Channel blocker recommended; avoid acetylcholinesterase inhibitors	[35,42]	5
*CHRNB1*	Fast-channel congenital myasthenic syndrome due to an acetylcholine receptor defect caused by pathogenic variants in *CHRNB1*	Pyridostigmine	Salbutamol or ephedrine	3,4-DAP		Fluoxetine or quinidine	Acetylcholinesterase inhibitors recommended; may require add-on second-line therapy	[60]	1
*CHRNB1*	Congenital myasthenic syndrome due to primary acetylcholine receptor deficiency caused by pathogenic variants in *CHRNB1*	Pyridostigmine	3,4-DAP	Salbutamol or ephedrine			Small number of reported cases; exploratory treatment with acetylcholinesterase inhibitor	[36]	1
*CHRND*	Slow-channel congenital myasthenic syndrome due to an acetylcholine receptor defect caused by a pathogenic variant in *CHRND*	Fluoxetine or quinidine				Pyridostigmine	Channel blocker recommended; avoid acetylcholinesterase inhibitors	[35,42]	2
*CHRND*	Fast-channel congenital myasthenic syndrome due to an acetylcholine receptor defect caused by pathogenic variants in *CHRND*	Pyridostigmine	Salbutamol or ephedrine	3,4-DAP		Fluoxetine or quinidine	Acetylcholinesterase inhibitors recommended; may require add-on second-line therapy	[59]	4
*CHRND*	Congenital myasthenic syndrome due to primary acetylcholine receptor deficiency caused by pathogenic variants in *CHRND*	Pyridostigmine	3,4-DAP	Salbutamol or ephedrine			Small number of reported cases; exploratory treatment with acetylcholinesterase inhibitor	[12]	2
*CHRND*	Congenital myasthenic syndrome due to defects in acetylcholine receptor clustering caused by pathogenic variants in *CHRND*	Pyridostigmine					Small number of reported cases; exploratory treatment with acetylcholinesterase inhibitor	[61]	1
*CHRNE*	Slow-channel congenital myasthenic syndrome due to an acetylcholine receptor defect caused by a pathogenic variant in *CHRNE*	Fluoxetine or quinidine				Pyridostigmine	Channel blocker recommended; avoid acetylcholinesterase inhibitors	[35,42]	11
*CHRNE*	Fast-channel congenital myasthenic syndrome due to an acetylcholine receptor defect caused by pathogenic variants in *CHRNE*	Pyridostigmine	Salbutamol or ephedrine	3,4-DAP		Fluoxetine or quinidine	Acetylcholinesterase inhibitors recommended; may require add-on second-line therapy	[59]	6
*CHRNE*	Congenital myasthenic syndrome due to primary acetylcholine receptor deficiency caused by pathogenic variants in *CHRNE*	Pyridostigmine	3,4-DAP	Salbutamol or ephedrine			Acetylcholinesterase inhibitors recommended; may require add-on second-line therapy	[34]	40
*CHRNE*	Congenital myasthenic syndrome with kinetic defect due to reduced ion channel conductance caused by pathogenic variants in *CHRNE*	Pyridostigmine					Small number of reported cases; exploratory treatment with acetylcholinesterase inhibitor	[62]	1
*COL13A1*	Congenital myasthenic syndrome due to collagen 13 defects caused by pathogenic variants in *COL13A1*	3,4-DAP	Salbutamol or ephedrine		Pyridostigmine		Small number of reported cases; exploratory treatment with β2 adrenergic receptor agonists and 3,4-DAP	[63]	2
*COLQ*	Congenital myasthenic syndrome due to endplate acetylcholinesterase deficiency caused by pathogenic variants in *COLQ*	Salbutamol or ephedrine				Pyridostigmine	β2 adrenergic receptor agonists recommended; avoid acetylcholinesterase inhibitors	[31]	35
*DOK7*	Congenital myasthenic syndrome due to defects in docking protein 7 caused by pathogenic variants in *DOK7*	Salbutamol or ephedrine				Pyridostigmine	β2 adrenergic receptor agonists recommended; avoid acetylcholinesterase inhibitors	[27]	40
*DPAGT1*	Congenital myasthenic syndrome due to a defect of glycosylation caused by pathogenic variants in *DPAGT1*	Pyridostigmine	3,4-DAP	Salbutamol or ephedrine			Acetylcholinesterase inhibitors recommended. May see additional benefit with addition of 3,4-DAP and salbutamol	[64]	7
*GFPT1*	Congenital myasthenic syndrome due to a defect of glycosylation caused by pathogenic variants in *GFPT1*	Pyridostigmine	3,4-DAP	Salbutamol or ephedrine			Acetylcholinesterase inhibitors recommended. May see additional benefit with the addition of 3,4-DAP and salbutamol; no effect on dystrophy expected	[38]	10
*GMPPB*	Congenital myasthenic syndrome due to a defect of glycosylation caused by pathogenic variants in *GMPPB*	Pyridostigmine	3,4-DAP	Salbutamol or ephedrine			Acetylcholinesterase inhibitors recommended. May see additional benefit with the addition of 3,4-DAP and salbutamol; no effect on dystrophy expected	[65]	6
*LAMB2*	Congenital myasthenic syndrome due to laminin β2 deficiency caused by pathogenic variants in *LAMB2*	Salbutamol or ephedrine					Small number of reported cases; exploratory treatment with β2 adrenergic receptor agonists	[66]	1
*LRP4*	Congenital myasthenic syndrome due to defects in low-density lipoprotein receptor-related protein 4 caused by pathogenic variants in *LRP4*	Salbutamol or ephedrine				Pyridostigmine	Small number of reported cases; exploratory treatment with β2 adrenergic receptor agonists	[67]	2
*MUSK*	Congenital myasthenic syndrome due to defects in MuSK caused by pathogenic variants in *MUSK*	Salbutamol or ephedrine				Pyridostigmine	Small number of reported cases; exploratory treatment with β2 adrenergic receptor agonists	[68]	11
*MYO9A*	Congenital myasthenic syndrome due to a defect in Myosin 9A caused by pathogenic variants in *MYO9A*	Pyridostigmine					Small number of reported cases; exploratory treatment with acetylcholinesterase inhibitors	[69]	2
*PLEC1*	Congenital myasthenic syndrome due to plectin deficiency caused by pathogenic variants in *PLEC1*	Pyridostigmine					Small number of reported cases	[70]	2
*PREPL*	Congenital myasthenic syndrome due to pathogenic variants in *PREPL* that predict reduced filling of synaptic vesicles with ACh	Pyridostigmine					Small number of reported cases; acetylcholinesterase inhibitors possibly beneficial in infancy	[71]	2
*RAPSN*	Congenital myasthenic syndrome due to endplate rapsyn deficiency caused by pathogenic variants in *RAPSN*	Pyridostigmine	3,4-DAP	Salbutamol or ephedrine		Fluoxetine	Acetylcholinesterase inhibitors recommended. May see additional benefit with addition of 3,4-DAP and salbutamol	[72]	40
*SCN4A*	Congenital myasthenic syndrome due to a sodium channel 1.4 defect caused by pathogenic variants in *SCN4A*	Pyridostigmine	Acetazolamide				Small number of reported cases; exploratory treatment with acetylcholinesterase inhibitors. Acetazolamide may be helpful for periodic paralysis	[73]	3
*SLC18A3*	Congenital myasthenic syndrome due to a vesicular acetylcholine transporter defect caused by pathogenic variants in *SLC18A3*	Pyridostigmine					Acetylcholinesterase inhibitors may be useful for respiratory crisis	[12]	2
*SLC25A1*	Congenital myasthenic syndrome due to a mitochondrial citrate carrier defect caused by pathogenic variants in *SLC25A1*	Pyridostigmine	3,4-DAP				Small number of reported cases; exploratory treatment with acetylcholinesterase inhibitors	[74]	1
*SLC5A7*	Congenital myasthenic syndrome due to a choline transporter defect caused by pathogenic variants in *SLC5A7*	Pyridostigmine	Ephedrine				Acetylcholinesterase inhibitors recommended	[12]	4
*SNAP25B*	Congenital myasthenic syndrome due to a synaptosomal-associated protein 25 defect caused by pathogenic variants in *SNAP25B*	3,4-DAP					Small number of reported cases; exploratory treatment with 3,4-DAP	[75]	1
*SYT2*	Congenital myasthenic syndrome due to a synaptotagmin defect caused by a pathogenic variant in *SYT2*	3,4-DAP					Small number of reported cases; exploratory treatment with 3,4-DAP	[76]	1
*UNC13A*	Congenital myasthenic syndrome due to a mammalian unco-ordinated-13 protein defect caused by a pathogenic variant in *UNC13A*	3,4-DAP	Pyridostigmine				Small number of reported cases; exploratory treatment with 3,4-DAP	[77]	1
*VAMP1*	Congenital myasthenic syndrome due to a vesicle-associated membrane protein 1 defect caused by a pathogenic variant in *VAMP1*	Pyridostigmine					Small number of reported cases; exploratory treatment with acetylcholinesterase inhibitors	[78]	1

This review investigates the evidence for pharmacological treatment of CMS. In addition to pharmacotherapy, non-drug treatments may be appropriate in CMS. These again vary by subtype according to symptom or specific phenotypic presentation, but may include physiotherapy, monitoring of respiratory and bulbar function, respiratory support, and gastric feeding tube if required. As with any genetic disease, genetic counseling for the family may be warranted. This systematic review does not cover non-pharmacological treatments of CMS.

### Objectives of the systematic review

Our objectives in performing this review were to identify the available evidence relating to the effect of pharmacological treatment of CMS, to systematically assess the strength of the evidence according to the Centre for Evidence-Based Medicine's (CEBM) Oxford 2011 Levels of Evidence [16], and to link treatments to precise genetic information at the variant level or the precise subtype level where possible. This study is designed to be the first step in making linked genotypic and treatment information available for genomics and clinical databases and computer-based expert analysis systems.

### Methods

The systematic review was designed using Cochrane Collaboration methodology [17] where possible and levels of evidence were assessed using the CEBM Oxford 2011 Levels of Evidence [16]. All steps were performed by two independent reviewers (R.T. and H.L.) with regular consensus meetings. A third reviewer (G.B.) critically reviewed the selected evidence and resolved disagreements. P.M. reviewed the evidence and provided input into the generation of a computer-readable dataset for the treatabolome.

#### Types of participants and diseases considered

We considered evidence dealing with adults and children with a genetic diagnosis of CMS or one of its subtypes, adopting a broad definition of CMS as any genetic neuromuscular condition manifesting with fatigable weakness of skeletal muscle and apparent NMJ involvement [18]. We included participants with any level of severity, age of onset and level of penetrance of phenotypic features, provided the underlying genetic defect gave rise to an apparent CMS.

#### Types of evidence considered

We considered for inclusion in the full systematic review randomized or quasi-randomized controlled trials of pharmacological treatment for CMS with defined genetic subtypes. Where no evidence from such trials was available, we considered nonrandomized trials, observational studies, case series and case reports in order to increase the available evidence base, while noting that such studies fall lower on the CEBM scale. For these evidence types, we provide a table of the evidence (Supplementary File S1) and a narrative summary and summary tables in the results section. Non-pharmacological interventions were out of the scope of this review.

#### Outcome measures

We considered all appropriate outcome measures clearly indicating (positive or negative) response to treatment, including change in scores and measures of muscle strength, functional ability and endurance, and clinical examination results. Studies unambiguously claiming treatment response but without providing full details of outcome measures used were considered in the narrative summary.

### Search methods for identification of studies

#### Literature and trial database search

To discover the available evidence covering pharmacological treatment for any congenital myasthenic syndrome, we performed electronic searches of the literature using several databases. Search terms, keywords, and filtration strategies are presented in Table 1. First, to identify potential sources of evidence corresponding to the highest CEBM evidence levels, we searched for randomized controlled trials and systematic reviews using the Cochrane Central Register of Controlled Trials (CENTRAL) [19], the ClinicalTrials.gov [20] and EU Clinical Trials [21] registers, and the Centre for Reviews and Dissemination (CRD) database [22], which includes the Database of Abstracts of Reviews of Effects (DARE) and NHS Economic Evaluation Database (NHS EED). Second, we searched the PubMed database [23] using a combination of keywords designed to capture the widest possible range of relevant literature, restricting our search to English language publications from 1980 to the present. Third, after manually reviewing the evidence from 1 and 2 and discarding duplicates and out-of-scope articles, we manually reviewed and extracted further relevant articles from the reference sections of the included articles.

#### Data collection, extraction, and analysis

The authors independently screened titles and abstracts of the publications identified through the searches in an unblinded manner to assess eligibility for inclusion, examining the full-text publication where abstracts were insufficiently informative. Studies that did not meet the inclusion criteria described were excluded. Full-text manuscripts were obtained for all studies passing the preliminary screening and used for data extraction.

Characteristics of each study were captured in a standardized data extraction form. Extracted data included study characteristics and design, presenting the phenotype of study participants, number of participants, age distribution, treatment characteristics: type, dose, frequency, and duration of treatment; characteristics of the outcome measures; and affected gene and variant.

#### Assessment of risk of bias in included studies

Our systematic review did not uncover any randomized controlled trials or other Level 1 or 2 evidence linked to genetically confirmed CMS; therefore, we have not carried out a formal assessment of risk of bias. According to Cochrane methodology, we must assume a high risk of bias for all nonrandomized studies, and the CEBM grade is correspondingly low.

## Results

**Figure 2.**
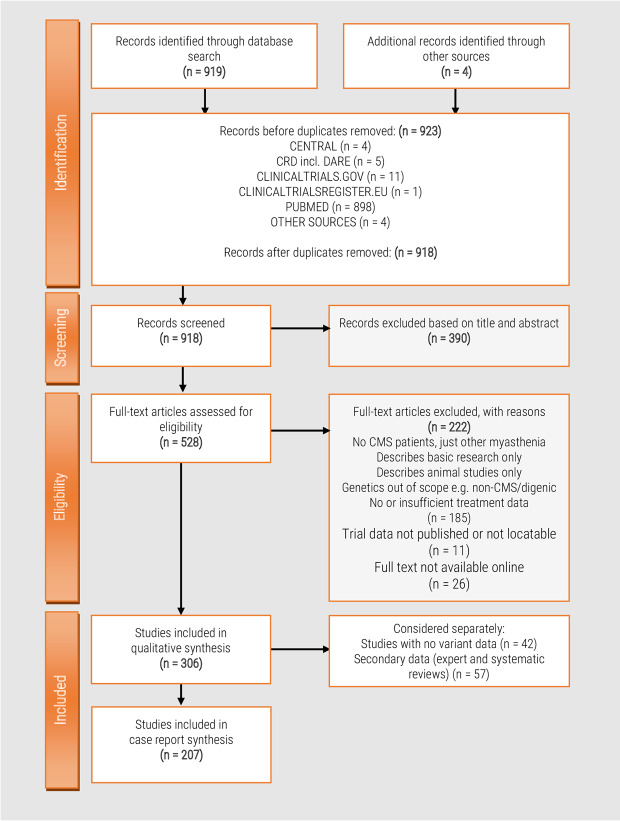
Systematic review flow chart. Flow diagram showing the literature evaluation process for the systematic review.

Our search methods resulted in an initial dataset of 918 studies to be reviewed. Three hundred and ninety were excluded as out-of-scope based on title and abstract: the majority of these studies either focused on a related disease that was out of scope of our review (usually autoimmune myasthenia gravis or Lambert-Eaton myasthenic syndrome) or were found to be animal studies or other basic science publications with no clinical data presented. A further 222 studies were excluded after review of the full text for the same reasons as above or because they did not present treatment data in sufficient detail for extraction. The full text of 26 studies was not available online. The systematic review process is summarized in Figure 2.

The details of the 306 remaining studies were extracted into a data extraction form for full analysis. We analyzed open-label studies, case reports, and case series (all of which report direct clinical observation of the patients) separately from systematic reviews and expert reviews (which summarize evidence in the light of expert opinion). Table 1 provides key recommendations and references from the extracted data and Supplementary File S1 provides further detail.

### Randomized controlled clinical trials

One 1991 study and one 1996 study [24,25] followed a randomized, double-blind placebo-controlled approach to 3,4-diaminopyridine administration in 4 and 11 clinically diagnosed CMS patients, respectively. Since no patient in these studies had a genetic diagnosis, we were unable to further classify the results and cannot take them into consideration in the treatabolome dataset, as it is not possible to establish whether the variation in response is due to differences in the underlying genetic defect (as the authors of the 1996 study themselves speculate). It is nevertheless worthy of note that two patients receiving placebo in the 1996 study had an apparent improvement of more than 20% in some evaluation scores. While this was less than the responders receiving the active treatment, it illustrates that the placebo effect may play a role even in clinical examinations and sounds an additional note of caution, particularly for the interpretation of individual case reports with little long-term follow-up.

We did not find any randomized controlled studies where the genetic defect was known and so we have no evidence of this level to include in the treatabolome dataset. All trials noted in the CENTRAL, clinicaltrials.gov, and EU trials register are listed as results not reported and were excluded from the final analysis.

### Systematic and literature reviews

We found one Cochrane systematic review of ephedrine treatment of myasthenia [26], which included CMS as well as acquired forms of myasthenia. This review provides a detailed narrative summary of the case reports analyzed, but found the evidence to be of ‘insufficient quality either to support or to discourage the use of ephedrine for these syndromes’. One systematic literature review summarizes the literature on pharmacological treatment of *DOK7* CMS [27] and concludes that treatment with salbutamol or ephedrine was beneficial in 65 of 69 patients, while other treatments trialed were beneficial in fewer cases, and in the case of AChE inhibitors might cause worsening. This is in line with the expert recommendations for *DOK7* treatment.

### Case reports, case series, and open-label trials

We found a total of 207 case reports, familial case reports, case series, and prospective open-label trials that provided information about treatment outcomes connected with genotype. Since the majority of these reports were not treatment trials but descriptions of novel genes or variants or the variant spectrum in a particular population, and were thus not originally designed to capture outcome measures in response to therapy, descriptions of outcome measures, treatment dose, duration, and response were usually very limited or absent. We provide the captured information in full in Supplementary File S1, and summarize the overall numbers in Table 1 and the evidence summary below. In the absence of treatment trials, this enables us to capture the number of published cases in which the response of a particular CMS type to therapy is positive, negative or equivocal, and may thus provide some insights into the weight of evidence that exists for a particular intervention. However, this evidence must be interpreted with caution. By their very nature, all such case reports have a high potential for subjectivity and bias. In addition, the treating clinician must remain aware that the response of patients even with identical causative variants may not always be the same, as many of these anecdotal cases illustrate.

### Expert opinion

Expert reviews differ from systematic and literature reviews in that the authors do not attempt to systematically capture all evidence in a format for analysis but rather to critically assess the published evidence in the light of their own experience and expert opinion. We list the reviews discovered in full in Supplementary File S1, but since expert opinion evolves over time, we have restricted our analysis to reviews published in the last 3 years. This includes two recent reviews specifically focusing on treatments for CMS [13,14] as well as many comprehensive summaries of the current state of CMS knowledge from expert centers with many years of experience in these rare conditions [12,28].

### Evidence summary

Our systematic review revealed that all known CMS types have received pharmacotherapeutic intervention of some kind. As is evident from Table 1, individual CMS types differ substantially in frequency and this is reflected in the weight of evidence available, with only a handful of publications covering the most recently discovered and rarest subtypes, while the more common subtypes each have numerically more substantial evidence to support treatment. The majority of patients receive either AChE inhibitors or β_2_ adrenergic receptor agonists as first-line treatment. AChE inhibitors such as pyridostigmine are commonly used in patients with AChR deficiency, most of which are caused by biallelic mutations in the *CHRNE* gene, while they should be avoided in patients with *DOK7* and *COLQ* defects, where they may be ineffective or may cause clinical worsening [29–31]. *DOK7* patients have been enrolled in several case series and open-label trials where they received β_2_ adrenergic receptor agonists (ephedrine, salbutamol) as off-label treatment with substantial benefit [32,33]. Recently, an increasing number of case studies report on the use of β_2_ adrenergic receptor agonists more generally, either as first-line therapy or as adjunctive treatment when the first-line therapy does not achieve the desired level of benefit alone [34]. Unlike AChE inhibitors, these drugs may take a period of weeks or months for therapeutic benefit to be fully realized [14]. 3,4-diaminopyridine is also used more frequently as adjunctive therapy than as the first-line treatment [13], except in some CMS types (see Table 1). Other drug treatments are only applied to small groups of patients with specific genetic defects based on assumptions derived from experimental models, e.g. channel blockers such as quinidine and fluoxetine in slow-channel CMS [35], where specific, dominantly acting missense mutations in any of the four AChR subunit genes (*CHRNE*, *CHRNA1*, *CHRNB1*, and *CHRND*) result in prolonged duration of channel opening, or the use of 3,4-diaminopyridine to increase acetylcholine quantal release in presynaptic CMS.

There is even less published information on the long-term outcomes of CMS therapies, yet patients typically require medication over many years, possibly life-long. In the more common CMS types, there have been some larger retrospective case series published where longer-term follow-up has been possible [36–38]. However, the majority of case reports only cover short-term response around the time of diagnosis, and further retrospective studies over a longer timeframe would be of value in this regard.

### Genetic confirmation

Our review identified 249 case reports/series that described pharmacological treatment of patients with a clinically and physiologically diagnosed CMS, 207 of which also had genetic confirmation in one of the known CMS causative genes. Our starting date for the data collection was 1980, a date that enabled us to capture the growing understanding of CMS pathophysiological mechanisms and treatments but that predates the first genetic confirmation of CMS by more than 10 years. Some of the early reports therefore did not provide genetic details defining the underlying defect, but the variant-level evidence provided in the 207 publications, covering over 1000 patients, can be included in the evidence for the treatabolome database. While attempts have been made to uncover diagnostic clues including EMG results and phenotypic hallmarks that, in the absence of a genetic test, result may still hint at specific molecular defects, and these are summarized in many recent reviews [39,40], phenotypic variability and overlap between CMS subtypes is such that a molecular diagnosis is considered the only reliable confirmation. Our review provides additional evidence to support the generally accepted position that optimal treatment does indeed vary by CMS type; and thus, knowledge of the exact genetic defect is crucial for making the correct treatment decision, identifying the most beneficial medication and avoiding potentially detrimental effects [40]. A logical conclusion is therefore that all patients with clinically suspected CMS should undergo genetic testing as a priority, if necessary with next-generation sequencing technology: this is a widely held aim for all genetic diseases but is particularly pressing in those that are treatable.

## Discussion

Our systematic review found no randomized controlled trials of pharmacological treatment and no CEBM level 1 or 2 evidence for any genetically determined congenital myasthenic syndrome. This was not an unexpected result, given the rarity of the condition and the fact that most pharmacological treatments for CMS are commonly available drugs prescribed in an off-label manner after rational selection based on their putative ability to correct the specific defect of neuromuscular transmission. While this means that there is a lack of evidence meeting the CEBM's higher level evidence categories, the review clearly shows that there is a substantial body of both expert opinion and case-based analysis available and that in many cases, significant efforts have been made to gather substantially sized cohorts for prospective or retrospective analysis [37,38,41,42]. This demonstrates that it is valuable to systematically assess and categorize the evidence that does exist: clinicians make daily treatment decisions that involve giving patients with CMS a pharmacological therapy, and providing better access to the evidence in support of those decisions is not only of practical benefit to those making the prescribing decisions, but also provides a gap analysis and guidance towards possible future options for clinical trials.

Even with comprehensive genetic testing, ∼20–40% of patients with CMS remain genetically undiagnosed [39]. Here, our systematic review cannot provide high-quality evidence for treatment decisions. Nevertheless, expert opinion generally favors a continuation of the historically applied principle of trialing treatments already known to be effective in other subtypes, following clinical decision trees based on phenotypic presentation and clinical insights into potential mechanisms [43]. A similar approach applies where there is an urgent treatment need while genetic results are pending, such as in cases of respiratory crisis and intensive care treatment, since the beneficial effect of the correct treatment has the potential to dramatically improve outcomes.

Most of the treatments used in CMS are based on a scientific rationale derived from a detailed understanding of the molecular and cellular pathophysiology of the neuromuscular junction that has been tested in cell and animal models [44]. In humans, widely available drug treatments were applied off-label based on scientific rationale (e.g. AChE inhibitors to increase availability of acetylcholine in the synaptic cleft) or serendipitous discovery (e.g. ephedrine, which was found to offer symptomatic relief in autoimmune myasthenia gravis) long before genetic confirmation became available. Some of these drugs have been the mainstay of CMS care in expert centers for over 30 years [45]. As our review confirms, randomized, placebo-controlled trials are almost entirely lacking, and clinical evidence is derived from a limited number of prospective and retrospective case series and a much larger number of individual and familial case reports, which have resulted in the generation of consensus opinion recommendations and expert reviews. Given the small number of individuals affected by any CMS type, and especially the rarest types, it is unrealistic to expect that randomized controlled trials are a likelihood in most cases. However, open-label studies where a patient is more systematically assessed using validated outcome measures before and after treatment might be a more realistic possibility, as is the publication of larger retrospective studies following the examples of some of the case series assessed here.

### Towards a treatabolome

Using the example of CMS, the systematic review presented in this paper shows that a wealth of knowledge linking therapeutic options for rare diseases directly with the genotype is potentially available to clinicians and geneticists. However, such knowledge is embedded in expert centers rather than in general practice, and while some of it makes its way into scientific publications as captured in this review, it is not readily available to clinicians at the moment they are confronted with a particular patient, as they cannot be expected to perform a literature review on the fly or to be an expert in every one of the more than 7000 currently known rare diseases.

We therefore propose the development of a new curated knowledge base that links genetic variants with therapeutic options in an interoperable form that facilitates incorporation into genomic analysis environments and clinical decision-support systems. This database, which we call the *treatabolome*, will contain a catalog of treatable genes/variants and associated treatment strategies for the diseases covered. The treatabolome will include all the relevant information for flagging the gene/variant in appropriate circumstances and providing evidence details to allow the clinician to evaluate whether the treatment should be further investigated.

The need for a regularly updated database of this kind is further increased by the dynamic nature of treatment recommendations, which may change over time as information evolves based on additional clinical experience and new gene discoveries. One reason for these changes is the growth over the past decade of NGS technologies that have dramatically increased the number of diagnosed rare diseases [46]. Its welcome expansion within healthcare systems worldwide will further increase diagnostic rates for both known and novel rare genetic disorders by making it easier to detect the genetic defect through non-targeted screening across the whole genome, even in cases where symptoms are unspecific or atypical and the correct diagnosis was not suspected by the geneticist or the clinician who ordered the test. The early and accurate diagnosis that NGS facilitates is of particular benefit in cases where dietary or enzyme replacement treatment from birth can slow or prevent deterioration, as is the case for several rare metabolic disorders and vitamin- and cofactor- deficiencies, and in cases where sudden and potentially fatal crises can arise in early childhood, including channelopathies and the episodic apnea that is a feature of some types of CMS. In our experience with neuromuscular disorders generally and CMS more specifically, the initiation of treatment is often delayed or treatment opportunities may be missed completely with negative consequences for the patients. While a correct interpretation of NGS results and an accurate molecular diagnosis is relevant for all families with rare genetic disease to support clinical decision-making and counseling, it is even more important where effective treatments are available that can be initiated upon reaching a diagnosis.

**Figure 3.**
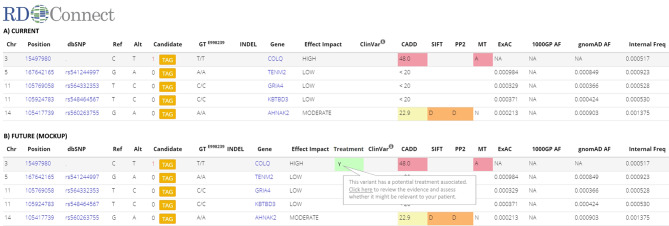
Mock-up of integration of treatabolome into the analysis system. (**A**) A section of the current analysis results interface in the RD-Connect Genome-Phenome Analysis Platform, which includes a range of variant-level information that helps the user to assess which of the candidate variants is most likely causative. (**B**) The way it would be possible to incorporate an additional column into the results interface to show the clinical end-user that one of the variants they are assessing has a potential treatment associated, enabling them to evaluate the evidence base to decide whether it is appropriate for the patient in question.

Motivated by these considerations, the CMS treatabolome will make variants in CMS-associated genes readily visible to non-disease-expert users of genetic databases, NGS analysis platforms, and gene-based decision systems, and flag up their potential treatment relevance and the existing evidence supporting it. It will be possible to incorporate the data into such systems through application programming interfaces or web services as envisaged in Figure 3 in order to highlight it to the end-user during use of the system, for example, at the time candidate variants are being assessed for pathogenicity. This will allow variants in treatable genes to be prioritized for timely follow-up and interpretation in the clinical and phenotypic context.

In terms of content and structure, the treatabolome will include items such as inheritance (recessive or dominant); penetrance (are there mutation carriers that do not develop the symptoms); severity (mild, severe/disabling, and life-limiting); age of onset (neonatal, childhood, and adult), and associated ontological terms from the Orphanet nomenclature and human phenotype ontology. Each genetic entry is connected to information about the treatment, such as treatment type (medication or other, e.g. diet and supplement); evidence (medication licensed for this gene/indication; unlicensed but in clinical guidelines); effect size of the treatment (curative, symptom-free, ameliorating symptoms, and extending lifespan); treatment indicated for asymptomatic (i.e. preventive) or symptomatic stages or specific symptoms/stages only; and a link to the relevant literature/resource.

The development of the treatabolome poses many challenges, especially with regard to curation, and will require expert input from both the clinical domain and the computational and data stewardship domain. Even this CMS systematic review, which started off with nearly 1000 studies to evaluate, covers only a small fraction of the currently known rare diseases and known causative variants, and the number is constantly growing, meaning that the database will need regular updates to remain relevant. Nevertheless, major online databases requiring constant curation such as Orphanet [47], ClinVar [48], LOVD [49], and others have run successfully in this domain for many years thanks to buy-in and substantial curation efforts from community experts, while international initiatives such as the International Rare Diseases Research Consortium [50], the Global Alliance for Genomics and Health [51], and RD-Connect [52] have shown that it is possible to bring expert working groups together internationally to achieve specific goals that can be transformational for the field. The same must be done for the linkage of variant information with treatment options: investment into the curation effort from the clinical and domain experts must be paired with data stewardship and interoperability expertise to generate a dataset that is compliant with the FAIR principles of findability, accessibility, interoperability, and reusability [53] and that has buy-in from the expert community.

The information on CMS treatments linked to the underlying CMS genetic defects that we provide here will be used to create a limited, pilot version of the treatabolome, which will be both interoperable and machine-readable [54] and will be suitable for incorporation into genetic databases and NGS analysis systems as guidance for end-users. The treatabolome concept will be applied to all rare neuromuscular disorders in future studies and extended to other rare genetic diseases as required, in particular under the auspices of the European Reference Networks, which provide a strategic framework for sharing of expertise in rare disease [55].

It is important to reiterate that the CMS treatabolome is not designed to replace clinical judgment by the experienced neuromuscular specialist. Instead, by flagging up potentially treatable conditions and providing immediate access to the evidence behind the result and the quality of the given evidence, it will assist the identification of treatable patients and support treatment decisions by better informing the treating clinician.

## Conclusion

Congenital myasthenic syndromes are very rare and highly heterogeneous, which makes traditional clinical trials to determine the efficacy and safety of medications difficult or impossible. This systematic review draws on small case series and case reports that form the basis of expert opinion indicating that highly effective drug treatments for various CMS subtypes exist, particularly AChE inhibitors and β_2_ adrenergic receptor agonists. Many CMS patients remain undiagnosed for years and treatment is delayed or may not be initiated even after exome sequencing if variants in CMS-associated genes are overlooked, have not been assigned as pathogenic in relation to the phenotype, or are not appreciated for their treatment relevance. This systematic review summarizes current knowledge about CMS treatments in relation to the underlying genetic defects and builds the foundation for an interoperable knowledgebase — the treatabolome — that will be used to support the identification of treatable variants at the time of diagnosis in genomic analysis systems such as RD-Connect. The treatabolome concept and bioinformatic scaffold will be rolled out more broadly to other rare genetic conditions through international collaborative efforts such as Solve-RD and the European Reference Networks.

## Summary

- A significant number of rare diseases are treatable with marketed therapies or commonly available drugs, but ensuring that patients receive the appropriate treatment in a timely manner remains challenging and requires new technologies suitable for the genomic era to support clinicians in accessing the relevant information.
- Very little high-level evidence exists for the drug treatment of congenital myasthenic syndromes (CMS), but an increasing body of expert opinion based on clinical evidence suggests that highly efficacious treatments do exist for the majority of patients and that treatment must be tailored to the underlying genetic defect. Here, we link genotype with treatment information for all CMS subtypes based on a systematic review of over 200 publications on more than 1000 patients.
- While expert opinion and systematic reviews may provide guidance and better access to evidence, finding the best treatment for an individual patient involves complex decision processes that remain the responsibility of an experienced clinician. Further prospective and controlled studies of treated CMS patients are required to provide better evidence for the long-term safety and efficacy of medications commonly used off-label.
- Next-generation sequencing is increasingly used as a first-line diagnostic tool for patients with unspecific neuromuscular complaints or weakness, some of whom may be affected by CMS. To alert the treating geneticist or clinician about patients with this highly treatable condition at the time of reviewing NGS results, we will integrate the information from this systematic review into a computer-readable and interoperable knowledgebase, the treatabolome, and expand the concept to other rare diseases with the buy-in of domain experts.

## Abbreviations

3,4-DAP: 3,4-diaminopyridine
AChE: acetylcholinesterase
AChR: acetylcholine receptor
CEBM: Centre for evidence-based medicine
CMS: congenital myasthenic syndrome
NGS: next-generation sequencing
NMJ: neuromuscular junction
